# Correlation between MIC of econazole nitrate and clinical response in otomycosis

**DOI:** 10.3389/fcimb.2026.1820764

**Published:** 2026-05-22

**Authors:** Yanwen Sun, Danqing Liu

**Affiliations:** 1 Department of Medical Laboratory, The First Affiliated Hospital of Zhejiang Chinese Medical University(Zhejiang Provincial Hospital of Chinese Medicine), Hangzhou, Zhejiang, China; 2 Department of Otolaryngology, The First Affiliated Hospital of Zhejiang Chinese Medical University(Zhejiang Provincial Hospital of Chinese Medicine), Hangzhou, Zhejiang, China

**Keywords:** *Aspergillus*, drug susceptibility, econazole nitrate, fungal otitis externa, species distribution, treatment

## Abstract

**Introduction:**

Otomycosis is a common superficial fungal infection in otolaryngology, with an increasing incidence that severely affects patients’ quality of life. There are regional variations in the distribution of dominant pathogenic fungi. This study was designed to determine the distribution of pathogenic fungi causing otomycosis in the local region, evaluate the *in vitro* susceptibility of predominant strains to econazole nitrate (a commonly used antifungal agent), and preliminarily investigate the correlation between drug susceptibility and clinical treatment duration, so as to fill the gap in regional research in this field.

**Methods:**

Ear secretion specimens from 196 patients with otomycosis were collected at the Department of Otolaryngology of Zhejiang Provincial Hospital of Chinese Medicine from December 2022 to December 2024 for isolation, culture, and identification of pathogens, along with drug sensitivity testing. Clinical data from 149 patients with complete follow-up records were combined to analyze the correlation between MIC and treatment duration.

**Results:**

Among the 196 patients, *Aspergillus* was the predominant pathogen, accounting for 85.10% of which *Aspergillus terreus * (*A. terreus*) was the dominant species, accounting for 68.88%. Significant interspecies differences in MIC of econazole nitrate were observed among different *Aspergillus* strains (P < 0.05). The clinical course in patients infected with *A. terreus* (3–90 days) and *Aspergillus niger* (*A. niger*) (7–60 days) was significantly positively correlated with their corresponding MIC (0.2-12.5 μg/mL and 0.59-37.5 μg/mL, respectively).

**Conclusion:**

The pathogenic spectrum of otomycosis in this region is unique, with *A. terreus* as the main pathogen. There are interspecies differences in the *in vitro* sensitivity of *Aspergillus* to econazole nitrate, and the elevated MIC values were significantly associated with prolonged time to clinical improvement in patients infected with *A. terreus* and *A. niger*.

## 1 Introduction

Otomycosis is a common superficial fungal infection in otolaryngology, particularly prevalent in warm and humid regions (Singh Gill et al., 2023). Globally, it accounts for approximately 9%-30% of external auditory canal infections (Mofatteh et al., 2018; Bojanović et al., 2023), with cases reported in Africa, Asia, Europe, and the Americas. In recent years, its incidence has shown an upward trend, making it a significant public health issue affecting global health (Ahmadi et al., 2023; Sathi et al., 2023; Diop et al., 2025). Clinical manifestations primarily include ear itching, otorrhea, aural fullness, and hearing loss, with some patients also experiencing otalgia, tinnitus, and other discomforts. Although the disease typically presents as a superficial infection confined to the external auditory canal, delayed diagnosis and inadequate treatment can lead to progression, resulting in severe complications such as tympanic membrane perforation, suppurative otitis media, and mastoiditis. Furthermore, in immunocompromised patients, the infection may spread systemically, imposing a substantial clinical burden and significantly impairing patients’ quality of life (Song et al., 2014; Kiakojuri et al., 2021). Therefore, early intervention and effective treatment of otomycosis are of great clinical significance. Currently, the diagnosis of otomycosis relies on the combination of otoscopic examination and mycological evidence. Otoscopy directly reveals white, yellow, or black fungal plaques adherent to the skin surface of the external auditory canal. Mycological tests, including fungal culture and microscopic identification of hyphae and spores, serve as the gold standard for definitive diagnosis (García-Agudo et al., 2011; Abraham, 2024). These tests not only validate the presence of fungal infection but also provide crucial insights for subsequent pathogen typing and the formulation of targeted treatment strategies, laying the foundation for personalized clinical management.

Previous studies have indicated that *Aspergillus* and *Candida* are recognized as the main pathogenic genera (Vennewald and Klemm, 2010; Zhang et al., 2020; Gu et al., 2022). However, the specific distribution of pathogenic fungi exhibits significant regional differences, and this heterogeneity may be associated with various factors such as climatic conditions, population living habits, and medical medication levels in different regions (Westby et al., 2020; Alshahni et al., 2021; Du et al., 2025). In studies conducted in southern Iran (Javidnia et al., 2022) and Wuhan, China (Chen and Zhao, 2024), *Aspergillus* was the dominant genus; in contrast, a study in Portugal showed that *Candida* accounted for the dominant position (Antunes et al., 2022). Additionally, it has been discovered that the *Aspergillus* complex is not a single species but consists of multiple species with similar morphologies but significant genetic differences. Among them, *Aspergillus tubingensis*(*A. tubingensis*) and *Aspergillus welwitschiae*(*A. welwitschiae*) are more common, and the distribution proportions of these species also vary significantly in different regions (Kamali Sarvestani et al., 2022; Halvaeezadeh et al., 2023). The complexity of this pathogenic composition further increases the difficulty of clinical treatment and highlights the necessity of conducting localized epidemiological investigations on pathogenic fungi.

Currently, the clinical treatment of otomycosis is based on the core strategy of “local debridement combined with topical antifungal agents”, among which the azole antifungal agent such as clotrimazole and miconazole, due to their broad-spectrum antifungal activity, have become the most widely used first-line treatment drugs (Vennewald and Klemm, 2010; Li et al., 2025). As an important member of imidazole antifungal drugs, econazole nitrate is widely used clinically, and multiple clinical studies have confirmed its effectiveness in combined treatment regimens (Du et al., 2025; Li et al., 2025). However, with the widespread application of antifungal drug, the problem of drug resistance has become increasingly prominent, which has become an important factor affecting clinical treatment effects, leading to disease recurrence and refractoriness (Kiakojuri et al., 2021; Ito et al., 2023). Previous studies have confirmed that *Aspergillus* strains isolated from patients with otomycosis exhibit high minimum inhibitory concentration (MIC) to commonly used azole antifungal drugs such as clotrimazole (Roohi et al., 2023; Peng et al., 2024). Moreover, isolates from patients with recurrent infections often possess biofilm-forming ability, a trait that may contribute to the development of refractory otomycosis (Bojanović et al., 2022). Clinical observations have also indicated that the treatment course for otomycosis is influenced by the species of the causative fungus. Notably, studies have reported that infections caused by *Aspergillus* species require a significantly longer treatment duration compared to those caused by *Candida* species (Antunes et al., 2022).

In light of the rising prevalence of antifungal resistance and the regional variability in the distribution of pathogenic fungi, conducting local epidemiological surveillance of fungal pathogens, along with *in vitro* susceptibility testing against commonly used antifungal agents, is of significant clinical importance. Such efforts are essential for informing optimized treatment strategies, improving patient outcomes, and mitigating the risk of drug resistance. To date, there is no systematic drug sensitivity test research on the commonly used drug econazole nitrate in this region. Clinical medication mostly relies on empirical treatment, and the research on the correlation between MIC and clinical course is still relatively lacking. Therefore, this study aims to clarify the distribution characteristics of pathogenic fungi causing otomycosis in this region, focusing on analyzing the species composition and distribution patterns of *Aspergillus* fungi. Meanwhile, the differences in susceptibility of different *Aspergillus* species to econazole nitrate were determined through drug sensitivity tests. Furthermore, the potential association between MIC and patients’ treatment courses was explored, so as to provide a basis for the precise treatment of this disease.

## 2 Materials and methods

### 2.1 Main instruments and reagents

0.45% hypotonic saline (bioMérieux SA, Cat. No. C1371A01), econazole nitrate (Sangon Biotech Co., Ltd., CAS No. 24169-02-6), MH nutrient broth (Hangzhou Microbial Reagent Co., Ltd.), analytical grade dimethyl sulfoxide (DMSO; Sinopharm Chemical Reagent Co., Ltd., No. 30072418), Sabouraud agar medium (Wenzhou Kangtai Biotechnology Co., Ltd.), 1300 Series Class II Type A2 Biosafety Cabinet (Esco Technologies, Singapore), Matrix-assisted laser desorption ionization-time of flight mass spectrometry (MALDI-TOF MS) (Bruker Corporation, Germany), GNP-9160 Digital Display Water-Jacketed Electric Incubator (Shanghai Jinghong Experimental Equipment Co., Ltd.), and MLS-3750 High-Pressure Steam Sterilizer (Tega SANYO Industry Co., Ltd.).

### 2.2 General information

Clinical data from 196 patients diagnosed with otomycosis at the Department of Otolaryngology of Zhejiang Provincial Hospital of Chinese Medicine from December 2022 to December 2024. This study was approved by the Ethics Committee of the First Affiliated Hospital of Zhejiang Chinese Medical University (Approval No. 2024-KLS-382-01). The data include gender, age, infection site, clinical symptoms, and underlying diseases (including hypertension, diabetes, neoplasms, otitis media, and external auditory canal eczema).The inclusion criteria for otomycosis were as follows: clinical manifestations including ear itching, fullness, pain, increased discharge, or hearing loss; otoscopic examination revealing erythema and swelling of the ear canal skin, along with fungal-like masses; and a positive fungal culture from ear discharge. Exclusion criteria comprised incomplete medical records, non-first-time treatment, inability to identify fungal species after culture, and mixed infections. The assessment of improvement after treatment was based on symptom relief, negative conversion of fungal smears, and normalization of otoscopic findings.

### 2.3 Isolation and culture of fungal strains

A total of 196 ear discharge samples were collected from patients. The samples underwent fungal isolation, cultivation, and identification using standard mycological protocols. Spores were gently scraped from the surface of *Aspergillus* colonies using sterile cotton swabs, stored in strain preservation tubes, and kept at -80°C for cryopreservation. The preserved *Aspergillus* strains were cultured: an appropriate amount of strains was picked with a sterile inoculating loop or needle and inoculated on Sabouraud agar medium using the “three-point inoculation method”. The Petri dish was sealed with parafilm, and incubated upside down at 28 °C for 5–7 days until the colonies matured.

### 2.4 Identification of *Aspergillus* strains

Morphological and mass spectrometric methods (MALDI-TOF MS) were employed for fungal identification. Initially, mature colonies grown on Sabouraud agar medium were subjected to morphological examination. This involved assessing colony purity to detect any contamination by other microorganisms and conducting preliminary species identification based on macroscopic characteristics, including colony size, color, texture, and growth rate (Azar, 2024). Subsequently, a direct formic acid extraction method was applied to hyphal tips for identification. In cases where this approach was unsuccessful, the fungus was cultured in nutrient broth until hyphal pellets developed. After collecting the mycelia, the supernatant was discarded by repeated centrifugation, and then a mixture of deionized water and absolute ethanol was added for treatment. After centrifugation to remove ethanol, the mycelia were dried at 37°C for 5–10 minutes. Then, 70% formic acid and an equal volume of acetonitrile were added in proportion to lyse the mycelia. After centrifugation, 1 μL of the supernatant was spotted on the target, covered with matrix solution and dried, and then detected by the instrument. Finally, the identification results were obtained through the mass spectrometry system.

### 2.5 Antifungal susceptibility testing

All *Aspergillus* isolates were tested using the broth macrodilution method according to the CLSI document M38-A3 (Alexander et al., 2017) proposed by the Clinical and Laboratory Standards Institute.

#### 2.5.1 Preparation of fungal suspensions

Conidia from *Aspergillus* isolates were collected using a cotton swab and suspended in 0.45% saline to achieve a turbidity equivalent to a 0.5 McFarland standard. A volume of 300 μL of this suspension was then added to 5.7 mL of inoculation broth and thoroughly mixed to obtain a fungal suspension at twice the final test concentration. For quality control, a 0.5 McFarland suspension of *Candida albicans* ATCC 14053 was prepared. Subsequently, 20 μL of this suspension was added to 2 mL of inoculation broth and mixed well. From this, 300 μL was further added to 5.7 mL of inoculation broth to prepare the working inoculum for the quality control strain.

#### 2.5.2 Preparation of antifungal working solutions

Dissolve 50 mg of econazole nitrate in 1 mL of analytical-grade DMSO to prepare a drug stock solution at a concentration of 5×10^4^μg/mL. For *Aspergillus niger* (*A. niger*) susceptibility testing: Pipette 90 μL of the stock solution into a test tube, add 7.41 mL of broth, and mix thoroughly to prepare a working solution with a total volume of 7.5 mL and a concentration of 600 μg/mL. For other *Aspergillus* species: Pipette 20 μL of the stock solution into a test tube, add 4.98 mL of broth to prepare a working solution with a total volume of 5 mL and a concentration of 200 μg/mL. In the antifungal susceptibility test for *A. niger*, the DMSO concentration corresponding to the highest antifungal drug concentration is 0.6%, whereas it is 0.4% for the other *Aspergillus* species.

#### 2.5.3 Antifungal susceptibility testing procedure for different *Aspergillus* species

A set of 12 sterile test tubes (10 mL capacity) was prepared for each isolate. Initially, 0.5 mL of broth was added to each tube. Subsequently, 0.5 mL of the 2 μg/mL econazole nitrate solution was introduced into the first tube and mixed thoroughly. A two-fold serial dilution was performed by transferring 0.5 mL from the first tube to the second tube, mixing well, and continuing this process sequentially through the tenth tube. Finally, 0.5 mL of the mixture was discarded from the tenth tube to maintain equal volumes. Following the drug dilution, 0.5 mL of the twice-concentrated fungal inoculum suspension was added to tubes 1 through 10. Tube 11 served as the positive growth control, receiving 0.5 mL of the fungal inoculum suspension without any antifungal agent. Tube 12 served as the negative sterility control, receiving 0.5 mL of sterile broth only. The concentration range of the drug against *A. niger* is 0.59-300 μg/mL, while the concentration range against other *Aspergillus* species excluding *A. niger* is 0.195-100 μg/mL. The final drug concentration range tested is detailed in **Table 1**. Each tube was securely capped with a silicone stopper and incubated at 35 °C. Fungal growth and MIC in each tube were recorded daily, and observations were maintained for 5 consecutive days.

**Table 1:** Econazole nitrate concentration gradient.

Species	Concentration(μg/mL)											
	1	2	3	4	5	6	7	8	9	10	11	12
*A. niger*	300	150	75	37.5	18.75	9.38	4.69	2.34	1.17	0.59	Positive	0
Other *Aspergillus*	100	50	25	12.5	6.25	3.125	1.563	0.781	0.391	0.195	Positive	0

### 2.6 Statistical analysis

Statistical analyses were performed using GraphPad Prism 6 and SPSS 25.0 software. Clinical data of patients with otomycosis, the distribution of pathogenic fungi, and antifungal susceptibility results were evaluated using the Kruskal-Wallis test, as well as Spearman or Pearson correlation coefficients, as appropriate. A *p*-value < 0.05 was considered statistically significant.

## 3 Results

### 3.1 Clinical characteristics of patients

Among the 196 enrolled patients, a male predominance was observed, with males accounting for 64.80% (127/196) of cases compared to 35.20% (69/196) for females. Patient ages ranged from 11 to 77 years, with the majority being young and middle-aged adults between 19 and 40 years old, representing 80.61% (158/196) of the cohort. Unilateral ear involvement was more prevalent, occurring in 73.46% (144/196) of patients. The most frequently reported clinical symptom was aural fullness, experienced by 42.86% (84/196) of patients, followed by pruritus 32.14% (63/196), otorrhea 15.31% (30/196), hearing loss 14.29% (28/196), tympanic membrane perforation 13.78% (27/196), otalgia 9.18% (18/196) and tinnitus7.14% (14/196). Patients with comorbidities accounted for 18.88% (37/196) (**Table 2**).

**Table 2:** Clinical data of 196 patients with otomycosis.

Characteristics	Number	Percentage (%)	*P*-Value
Gender			
Male	127	64.80	< 0.001
Female	69	35.20	
Age range (years)			
0-18	7	3.57	< 0.001
19-40	158	80.61	
41-60	26	13.27	
>60	5	2.55	
Infection status			
Unilateral	144	73.46	< 0.001
Bilateral	46	23.47	
No record	6	3.06	
Clinical symptoms			
Aural fullness	84	42.86	< 0.001
Pruritus	63	32.14	
Otorrhea	30	15.31	
Hearing loss	28	14.29	
Tympanic membrane perforation	27	13.78	
Otalgia	18	9.18	
Tinnitus	14	7.14	
Underlying disease			
Yes	37	18.88	< 0.001
No	159	81.12	

**Figure 1:**
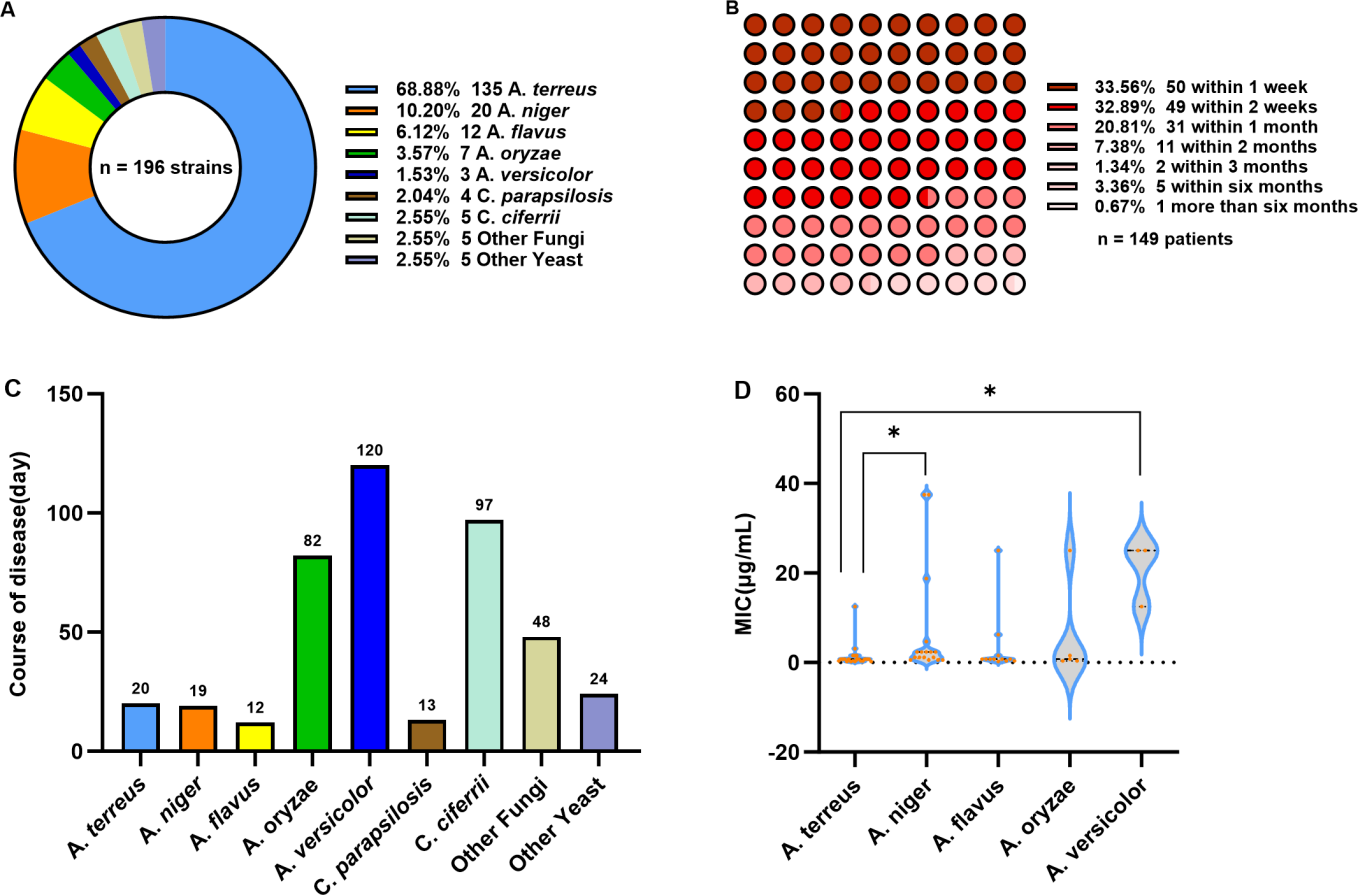
Pathogenic characteristics and clinical treatment of patients with otomycosis. **(A)** Distribution of pathogenic fungi in 196 patients with otomycosis. **(B)** Overall clinical treatment duration characteristics in 149 patients with otomycosis. **(C)** Clinical course analysis of patients infected with different fungal species. **(D)** Susceptibility analysis of different *Aspergillus* species to Econazole Nitrate, comparing the MIC of Econazole Nitrate against *A. terreus*, *A. niger*, *A. flavus*, *A. oryzae*, and *A. versicolor*, showing statistically significant differences between *A.* t*erreus* and both *A. niger* and *A. versicolor*. * P < 0.05.

*Aspergillus* species were the predominant pathogens isolated, accounting for 85.10% of cases. Among these, *Aspergillus terreus* (*A. terreus*) was the most common, representing 68.88% (135/196) of all isolates, followed by *A. niger*, *Aspergillus flavus* (*A. flavus*), and others (**Figure 1A**).

A total of 149 patients had complete follow-up records in this study. The proportion of patients who showed improvement within 1–2 weeks was the highest (66.45%), followed by those who improved within 1 month (20.81%) (**Figure 1B**). Among patients with different fungal infections, those infected with *Aspergillus versicolor* (*A. versicolor*) had the longest mean time to improvement (120 days), followed by those infected with *Candida ciferrii* (*C. ciferrii*) (97 days) and *Aspergillus oryzae* (*A. oryzae*) (82 days). In contrast, *A. flavus*, *Candida parapsilosis* (*C. parapsilosis*), *A. niger*, and *A. terreus* had shorter mean time to improvement, with values of 12, 13, 19, and 20 days, respectively (**Figure 1C**).

### 3.2 Correlation between fungal species and patients’ characteristics and manifestations

No significant differences were observed among fungal species in terms of gender distribution (*P* > 0.05), age distribution (*P* > 0.05) or infection status (*P* > 0.05). However, the presence of underlying diseases differed significantly across groups (*P* < 0.001). Specifically, underlying diseases were present in 81.82% (9/20) of *A. niger*, 66.67% (2/3) of *A. versicolor*, 50.00% (6/12) of *A. flavus*, and 42.86% (3/7) of *A. oryzae*, compared with only 9.76% (12/135) of *A. terreus* (**Table 3**).

**Table 3:** Basic characteristics of patients with otomycosis.

Parameter	*A. terreus*	*A. niger*	*A. flavus*	*A. oryzae*	*A. versicolor*	*C. parapsilosis*	*C. ciferrii*	Other fungi	Other yeast	*P*-Value
Number	135	20	12	7	3	4	5	5	5	
Gender										
Male	89	10	7	4	2	4	4	4	3	0.540
Female	46	10	5	3	1	0	1	1	2	
Age range (years)										
0-18	5	2	0	0	0	0	0	0	0	0.846
19-40	116	10	9	6	3	3	4	4	3	
41-60	13	6	2	0	0	1	1	1	2	
≥61	1	2	1	1	0	0	0	0	0	
Infection status										
Unilateral	95	18	10	5	2	2	4	5	3	0.280
Bilateral	36	1	1	2	1	2	1	0	2	
Underlying diseases										
Yes	12	9	6	3	2	1	2	2	0	<0.001
No	123	11	6	4	1	3	3	3	5	

Notably, specific symptoms were associated with particular fungal species. Infections caused by *A. niger* (45%) and *C. ciferrii* (42.86%) were frequently accompanied by tympanic membrane perforation. Patients infected with *A. flavus* (50%) and *A. oryzae* (60%) often presented with otorrhea. *A. versicolor* (75%) infections were commonly associated with hearing loss, while *C. parapsilosis* (60%) infections were frequently accompanied by pruritus (**Table 4**).

**Table 4:** Clinical manifestations of patients with otomycosis.

Parameter	*A. terreus*	*A. niger*	*A. flavus*	*A. oryzae*	*A. versicolor*	*C. parapsilosis*	*C. ciferrii*	Other yeast	Other fungi	*P*-Value
Number (n)	135	20	12	7	3	4	5	5	5	
Clinical manifestations (%)										
Tympanic membrane perforation	5.19	45.00	25.00	20.00	0.00	0.00	42.86	40.00	66.67	0.000
Otalgia	8.15	25.00	0.00	0.00	0.00	20.00	0.00	20.00	0.00	0.148
Hearing loss	13.33	10.00	0.00	20.00	75.00	0.00	28.57	0.00	66.67	0.001
Tinnitus	9.63	0.00	0.00	0.00	0.00	0.00	14.29	0.00	0.00	0.560
Otorrhea	5.19	30.00	50.00	60.00	25.00	40.00	14.29	40.00	66.67	0.000
Pruritus	34.81	10.00	33.33	40.00	25.00	60.00	0.00	60.00	33.33	0.118
Aural fullness	66.67	35.00	41.67	20.00	50.00	40.00	42.86	20.00	33.33	0.027

### 3.3 Antifungal susceptibility of *Aspergillus* to econazole nitrate

Given that *Aspergillus* species were the predominant pathogens isolated from the 196 patients with otomycosis, this study specifically evaluated their susceptibility to econazole nitrate. The MIC results for econazole nitrate against five *Aspergillus* species are presented in **Table 5** and Supplement 1. Kruskal-Wallis test revealed a statistically significant difference in MIC among the various *Aspergillus* species (*P* < 0.05). Subsequent pairwise comparisons indicated that the MIC for *A. terreus* were significantly different from those for both *A. niger* and *A. versicolor* (*P* < 0.05), while there were no statistically significant differences between the other groups (**Figure 1D**). Furthermore, *A. versicolor* exhibited the highest MIC_50_ (25 μg/mL) and MIC_90_ (25 μg/mL) among the tested species, followed by *A. niger* (2.34 μg/mL, 18.75 μg/mL). Notably, *A. niger* also demonstrated the widest range of MIC (0.59-37.5 μg/mL), followed by *A. flavus* (0.20-25 μg/mL).

**Table 5:** Antifungal susceptibility of *Aspergillus* species to econazole nitrate.

Species	Number(n)	MIC(μg/mL)			
		MIC_50_	MIC_90_	Range	*P*-Value
*A. terreus*	22	0.78	1.56	0.20-12.50	0.006
*A. niger*	17	2.34	18.75	0.59-37.5	
*A. flavus*	12	0.78	6.25	0.20-25	
*A. oryzae*	5	0.78	1.56	0.39-25	
*A. versicolor*	3	25	25	12.5-25	

### 3.4 Correlation between MIC and clinical course

**Figure 2:**
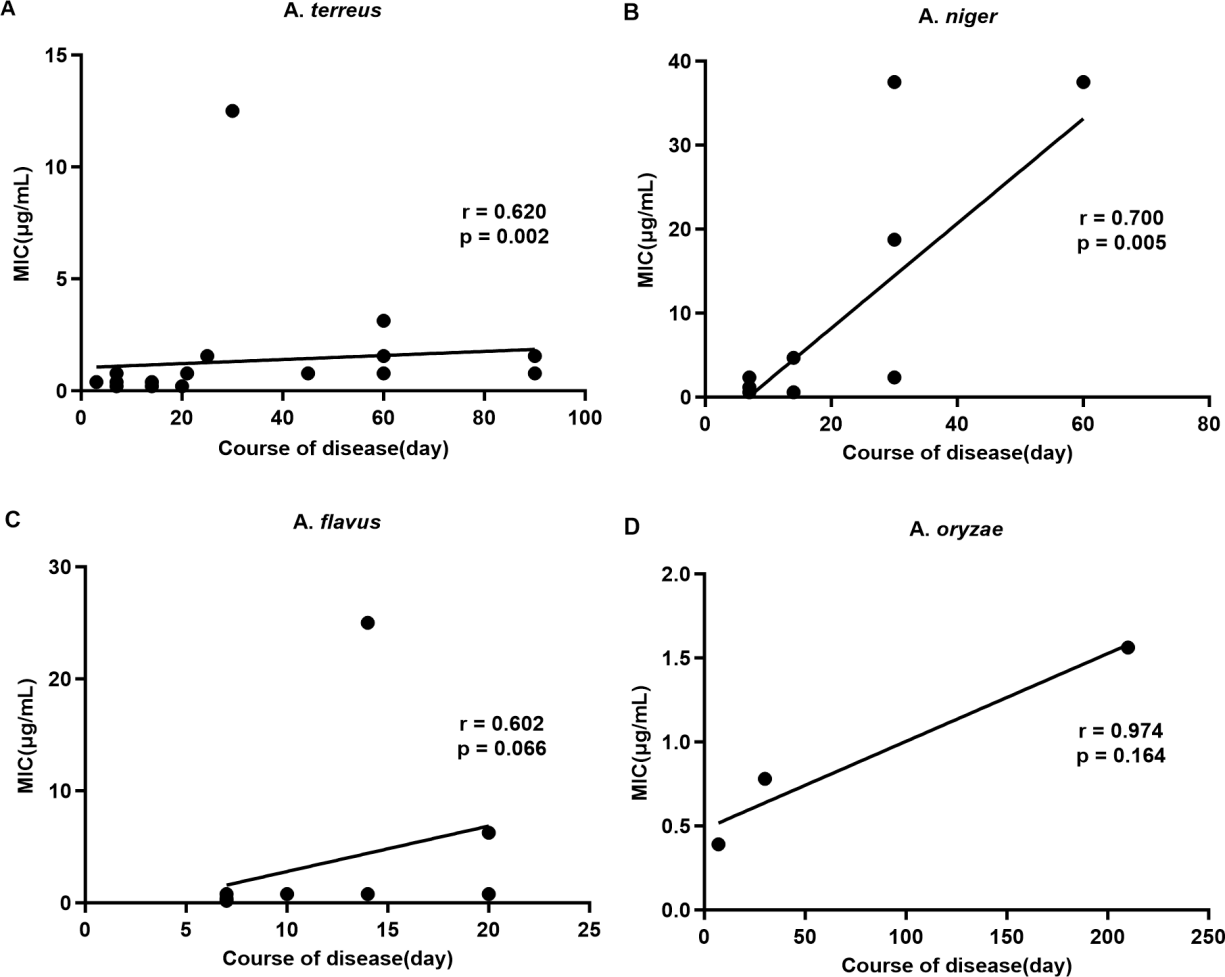
Correlation between the drug susceptibility of different *Aspergillus* species to Econazole Nitrate and the clinical course of the disease. **(A)** *Aspergillus terreus*. **(B)** *Aspergillus niger*. **(C)** *Aspergillus flavus*. **(D)** *Aspergillus oryzae*.

To further examine the correlation between the MIC of different *Aspergillus* species and the patients’ clinical course, Spearman or Pearson correlation analysis was conducted. The correlation coefficients between MIC and clinical course were 0.620 for *A. terreus*, 0.700 for *A. niger*, 0.602 for *A. flavus*, and 0.974 for *A. oryzae*. Statistical analysis was not performed for *A. versicolor* due to the limited number of cases. A significant positive correlation was observed between MIC and time to improvement in patients infected with *A. terreus* and *A. niger* (*P* = 0.002 and *P* = 0.005, respectively). Although positive correlations were also noted for *A. flavus* and *A. oryzae*, they did not reach statistical significance (*P* = 0.066 and *P* = 0.146, respectively) (**Figure 2**).

Furthermore, to control for potential confounding effects of gender, age, underlying diseases, and infection site, a multivariate logistic regression analysis was performed. The results indicated that after adjusting for these confounders, none of the variables included in the study demonstrated a statistically significant independent predictive value for the clinical course (*P* > 0.05) (**Table 6**).

**Table 6:** Correlation between clinical characteristics and disease course in otomycosis.

Characteristics	*A. terreus*	*A. niger*	*A. flavus*	*A. oryzae*
Gender				
Male	14	7	5	2
Female	8	7	5	1
Age (years)	29.82 ± 9.22	37.0 ± 16.0	34.9 ± 11.76	32.33 ± 4.51
Underlying disease				
Yes	3	5	5	1
No	19	9	5	2
Infection status				
Unilateral	17	14	10	2
Bilateral	5	0	0	1
MIC (μg/ml)	0.20-12.50*	0.59-37.5*	0.20-25	0.39-25
Disease course (days)	34.6 ± 29.6	20 ± 15.66	12.0 ± 5.27	82.33 ± 111.16

*P<0.05 for the correlation between MIC and disease course in Otomycosis (Spearman’s rank correlation).

## 4 Discussion

Currently, the specific pathogenesis of otomycosis remains unclear. Existing literature suggests that the development and progression of this disease are primarily regulated by a combination of environmental factors and host-related factors, with their synergistic interaction likely constituting a critical step in disease initiation. Among environmental risk factors, the warm and humid climate characteristic of tropical and subtropical regions is most prominent, providing optimal temperature and humidity for fungal growth and reproduction, thereby significantly increasing the risk of local fungal colonization (Singh Gill et al., 2023; Du et al., 2025). Host-related risk factors are diverse, primarily including impaired drainage of secretions due to the unique anatomical structure of the external auditory canal (e.g., narrow or curved canals), excessive cerumen secretion, local skin trauma within the ear canal, long-term use of occlusive hearing aids, dysbiosis following topical antimicrobial therapy, cross-infection from inadequately treated dermatophytosis, and compromised antifungal immunity associated with systemic immunodeficiencies (e.g., diabetes mellitus, long-term immunosuppressant use, acquired immunodeficiency syndrome, etc.) (Gohar et al., 2014; Abdelazeem et al., 2015). Furthermore, the age and sex distribution of otomycosis varies across different regions and populations. Analysis of clinical data from 196 patients in the present study revealed that while the disease can affect individuals across all age groups, it was predominantly observed in young and middle-aged adults between 19 and 40 years, accounting for 80.61% of cases. This finding is consistent with previous studies (Alarid‐Coronel et al., 2018; Ali et al., 2018; Sangaré et al., 2021), suggesting that this demographic may represent a high-risk group, potentially due to lifestyle habits and higher levels of activity. Concurrently, our study observed a significantly higher proportion of male patients compared to females, aligning with some prior reports (Abdelazeem et al., 2015; Tasić-Otašević et al., 2020). This gender disparity may be attributable to factors such as increased sebaceous and sweat gland secretion in males, as well as frequent ear cleaning practices that could disrupt the microecology of the external auditory canal, predisposing individuals to fungal infection. These observations provide a valuable reference for developing targeted preventive strategies for different populations.

The distribution characteristics of pathogenic fungi constitute a core premise for guiding the treatment of otomycosis, and the pathogenic spectrum varies across different regions due to differences in climate, environment, and population living habits. The results of this study showed that the main pathogenic fungi of otomycosis in this region were *Aspergillus*, accounting for 85.10%, among which *A. terreus* was the absolute dominant species, accounting for 68.88% of *Aspergillus* strains. This pathogenic distribution pattern is consistent with findings from a recent study in the Jingzhou region of China (Peng et al., 2024), suggesting that *A. terreus* may be the primary pathogenic fungus in the subtropical areas of southern China. However, compared with studies from other global regions, the pathogenic spectrum in our area shows distinct differences: in most regions worldwide, such as southern Iran (Javidnia et al., 2022) and northern China (Jing et al., 2022), the *A. niger* complex (e.g., *A. tubingensis*, *A. welwitschiae*) dominates among the causative agents of fungal external otitis (Nazari et al., 2025). This geographical heterogeneity further underscores the importance of conducting localized epidemiological investigations of pathogenic fungi (Stemler et al., 2023). Furthermore, this study explored the correlation between fungal species and clinical symptoms as well as complications. The results showed that typical clinical manifestations of otomycosis include persistent pruritus, otalgia, otorrhea, aural fullness, and hearing loss. When the disease progresses to involve the middle ear, severe complications such as tympanic membrane perforation may occur. Previous studies have reported that tympanic membrane perforation, as a severe complication of otomycosis, has an incidence rate as high as 6.75%-36.11%, and is associated with infections by specific species such as *A. flavus* and *A. tubingensis* (Kiakojuri et al., 2021; Javidnia et al., 2022). The present study further confirms a significant correlation between infections caused by *A. niger* and *C. ciferrii* and tympanic membrane perforation. Additionally, different pathogens tend to induce distinct clinical symptoms. For instance, infections with *A. flavus* and *A. oryzae* are more prone to causing otorrhea, while *C. parapsilosis* infections primarily present with pruritus. These findings suggest that the specific fungal species may modulate the clinical presentation of the disease by influencing the intensity of the local inflammatory response in the ear canal and the degree of tissue destruction. However, the underlying molecular mechanisms remain unclear, and further multicenter studies with larger sample sizes are needed for further exploration.

Econazole nitrate, an imidazole antifungal agent, is widely used in the treatment of otomycosis, and its efficacy is supported by relevant studies (Gülüstan et al., 2021). This drug primarily functions by inhibiting the synthesis of ergosterol and phospholipids in the fungal cell membrane, thereby disrupting the integrity and permeability of the membrane, leading to impaired fungal cell function and promoting apoptosis (Ghannoum and Rice, 1999; Harada et al., 2025). In the present study, clinical efficacy analysis revealed that the treatment duration for infections caused by *Aspergillus* species was significantly longer than that for *Candida* infections, which is consistent with a clinical observational study from Portugal (Nazari et al., 2025). Further analysis of treatment data for different *Aspergillus* species demonstrated significant variability in the time to clinical improvement among patients infected with various species. Notably, patients infected with *A. versicolor* experienced the longest treatment course. It is hypothesized that this may be related to the inherent growth characteristics of different *Aspergillus* species, variations in their drug susceptibility to econazole nitrate, and potential differences in biofilm-forming capacity. Previous studies have confirmed that fungal biofilm formation is closely associated with the development of refractory and recurrent otomycosis (Bojanović et al., 2022). Biofilms can reduce fungal susceptibility to antifungal agents, increasing treatment difficulty and prolonging the course of therapy. To further investigate the underlying factors contributing to these differences in treatment outcomes, antifungal susceptibility testing was performed. According to CLSI guidelines, the interpretation of MIC results relies on established clinical breakpoints. However, no CLSI breakpoints have been established for econazole against *Aspergillus* species to date. Therefore, the MIC obtained in this study cannot be categorized as susceptible, intermediate, or resistant, and we report them solely as raw MIC values. The results revealed significant interspecies differences in drug susceptibility. *A. versicolor* exhibited the highest MIC_50_ and MIC_90_, suggesting that this species has the lowest susceptibility to econazole nitrate among those tested. In contrast, *A. niger* displayed the widest range of MIC distributions, indicating substantial variability in susceptibility among different isolates of this species. It is noteworthy that despite the widespread use of azole antifungals in treating otomycosis, studies have reported that *Aspergillus* strains isolated from patients with otomycosis can exhibit high MIC to azoles such as clotrimazole (Halvaeezadeh et al., 2023), and some strains may even demonstrate resistance to newer triazoles like voriconazole and itraconazole (Jing et al., 2022).

Further analysis in this study revealed a significant positive correlation between clinical course and the MIC of econazole nitrate for the corresponding strains in patients infected with *A. terreus* and *A. niger*. This result provides a potential basis for translating *in vitro* susceptibility data into clinical therapeutic decisions, such as estimating the required treatment duration or assessing the need for treatment regimen escalation (Merad et al., 2021). However, validation through larger-scale statistical studies is necessary. Although a positive trend was observed between clinical improvement time and MIC for patients infected with *A. flavus*, *A. oryzae*, and *A. versicolor*, these correlations did not reach statistical significance. This lack of significance is potentially attributable to the relatively small sample sizes for these species, resulting in insufficient statistical power. Additionally, analysis of follow-up data from 149 patients in this study demonstrated that 66.45% of patients achieved clinical improvement within 1–2 weeks, 20.81% required up to one month for cure, and 12.75% needed an extended treatment course ranging from two months to six months. These findings indicate that while the majority of patients in our region achieve favorable outcomes through short-term standard treatment, a subset of cases show poor therapeutic response. This is presumably associated with drug resistance in specific strains. The emergence of antifungal drug resistance represents a multifaceted biological challenge driven by both intrinsic genetic adaptability and extrinsic environmental pressures. Classical mechanisms of azole resistance primarily involve alterations in the amino acid sequence of the drug target enzyme, lanosterol 14α-demethylase (encoded by ERG11 or CYP51), thereby diminishing the binding affinity between the drug and its target (Boyce, 2023). Additionally, fungi can actively reduce intracellular drug concentrations through the upregulation of membrane-associated efflux transporters that pump drugs out of the cell (Sipos and Kuchler, 2006). Another well-recognized contributor to fungal drug resistance is the biofilm growth mode; when fungi grow as biofilms, they exhibit a significantly enhanced drug-resistant phenotype compared to planktonic cells, which is attributed to the protective barrier formed by the biofilm matrix and altered metabolic activity within the biofilm community (Kowalski et al., 2020). Recent advances have illuminated additional layers of complexity underlying antifungal resistance. Exposure to antifungal agents triggers diverse cellular stress response pathways that promote fungal survival under pharmacological pressure. Furthermore, the remarkable genomic plasticity observed in fungal pathogens contributes to their evolutionary capacity to acquire and maintain resistance traits (Boyce, 2023; Lee et al., 2023). These multifaceted resistance mechanisms collectively pose substantial challenges to the clinical management of fungal infections and highlight the necessity of in-depth exploration of regional resistance profiles and their underlying molecular basis. Future research should focus on in-depth investigation of resistant species and further development of novel therapeutic strategies. Concurrently, the feasibility of combination therapy approaches warrants exploration. For instance, the combination of terbinafine and ketoconazole has demonstrated *in vitro* synergistic potential against certain *Aspergillus* species (Nosratabadi et al., 2023), which could provide additional evidence for optimizing the clinical management of otomycosis.

However, the MIC determined by fungal susceptibility testing still presents certain limitations in guiding clinical antifungal therapy. First and foremost, the MIC derived from *in vitro* susceptibility assays primarily reflects the *in vitro* inhibitory activity of antifungal agents, yet it cannot fully represent the fungicidal activity (minimum fungicidal concentration, MFC) or the actual therapeutic efficacy *in vivo*. A comparative study investigating the MIC and MFC of azole drugs against dermatophytes demonstrated that MFC were consistently higher than corresponding MIC (Hiruma et al., 2024). Second, the absence or inconsistency of standardized clinical breakpoints or epidemiological cutoff values (ECVs) compromises the clinical interpretability of MIC results. Previous studies have revealed that for certain fungal species, including *Candida glabrata* and *Candida krusei*, distinct susceptibility categorizations for echinocandin antifungals may arise from different detection methods. Moreover, the application of divergent interpretive criteria, such as clinical breakpoints versus ECVs, can lead to discrepant classification outcomes, thereby introducing uncertainty into MIC-guided clinical medication decisions (Lee et al., 2022). In addition, conventional MIC testing is primarily designed to evaluate the susceptibility of the currently isolated fungal strain, with limited capacity to predict future trends in antifungal resistance (Liu et al., 2018). Therefore, continuous surveillance of fungal susceptibility profiles across different geographic regions is warranted to inform evidence-based clinical management of fungal infections.

## 5 Conclusion

In conclusion, *A. terreus* was identified as the predominant pathogen causing otomycosis in our region. *In vitro* antifungal susceptibility testing revealed interspecies variation in the minimum inhibitory concentration (MIC) of econazole nitrate among *Aspergillus* species. Notably, elevated MIC were significantly associated with prolonged clinical improvement in patients infected with *A. terreus* and *A. niger*, suggesting the potential utility of susceptibility testing in the management of such cases. These findings underscore the importance of local epidemiological data combined with antifungal susceptibility profiles in guiding therapeutic strategies for otomycosis.

## Data Availability

The raw data supporting the conclusions of this article will be made available by the authors, without undue reservation.
